# Molecular Mechanisms Driving Metastatic Progression Within the Aged Tumor Microenvironment

**DOI:** 10.3390/ijms262311508

**Published:** 2025-11-27

**Authors:** Sudhir Kumar, Jagdish Chand, Preeti Sharma, Sudhakar Singh, Pukar Khanal, Hanish Singh Jayasingh Chellammal, Aamir Suhail, Sonam Mittal

**Affiliations:** 1 Department of Microbiology and Immunology, Emory University School of Medicine, Atlanta, GA 30322, USA; 2 Department of Pharmaceutical Chemistry, JSS College of Pharmacy, JSS Academy of Higher Education & Research, Ooty 643001, Tamil Nadu, India; 3 Department of Pharmacy, PSIT—Pranveer Singh Institute of Technology (Pharmacy), Bhauti, Kanpur 209305, Uttar Pradesh, India; 4 Emory Vaccine Center, Emory National Primate Research Center, Emory University, Atlanta, GA 30322, USA; 5 Department of Pharmacology and Chemical Biology, Emory University School of Medicine, Atlanta, GA 30322, USA; 6 Department of Pharmacology and Life Sciences, Faculty of Pharmacy, Universiti Teknologi MARA (UiTM), Puncak Alam 42300, Selangor, Malaysia; 7 The Gene Lay Institute of Immunology and Inflammation, Brigham and Women’s Hospital, Mass General Hospital, and Harvard Medical School, Boston, MA 02115, USA; 8 Department of Medicine, Washington University, St Louis, MO 63110, USA

**Keywords:** aging, tumor microenvironment, metastasis, cancer, cellular senescence, immune dysfunction, aging hallmarks, molecular mechanism

## Abstract

Metastasis remains the leading cause of cancer deaths, heavily influenced by aging-related biological processes. As global life expectancy increases, cancer incidence and progression complexity in older adults also rise, emphasizing the urgent need to understand how the aging tumor microenvironment (TME) promotes metastasis. This review explores the molecular and cellular mechanisms behind metastatic development in the aged TME, focusing on the combined impacts of cellular senescence, chronic inflammation (inflammaging), immune system decline, extracellular matrix (ECM) changes, and abnormal blood vessel growth. Aging causes an accumulation of senescent cells that secrete a range of cytokines, growth factors, and enzymes (SASPs), which remodel the ECM, making it stiffer and more degradable, and activate pro-metastatic pathways like TGF-β, STAT3, and MAPK, aiding processes like EMT and tumor invasion. Meanwhile, persistent low-grade inflammation attracts immunosuppressive cells, and immune decline hampers tumor surveillance, allowing cancer cells to evade immune detection. The aged TME also undergoes significant vascular and metabolic changes, such as abnormal angiogenesis and hypoxia, supporting the growth of more aggressive, treatment-resistant cancer clones and spreading metastases. These changes are driven by hallmarks of molecular aging, including telomere shortening, oxidative DNA damage, and epigenetic alterations, which lead to genetic instability and turn the aged stroma into fertile ground for metastasis. The review also discusses new therapeutic approaches, including senolytics, anti-inflammatory treatments, immune system rejuvenation, and metabolic strategies, highlighting the importance of age-specific models and precision medicine to enhance outcomes for the growing number of elderly cancer patients.

## 1. Introduction

Cancer remains the leading cause of death worldwide, significantly contributing to the global disease burden and loss of life each year. According to the World Health Organization, metastasis, the spread of malignant cells from the primary tumor to distant organs, accounts for most cancer-related deaths. However, its impact varies by tumor type. For example, metastatic disease causes nearly 90% of deaths in breast, lung, and colorectal cancers, while the rate is lower in certain hematologic malignancies and localized prostate cancers [1,2].

Each year, approximately 18 million people are diagnosed with cancer globally, and a substantial proportion eventually develop metastatic lesions, with the incidence ranging from about 6% in breast cancer to over 50% in lung and pancreatic cancers [3]. Among the most prevalent malignancies, breast, lung, colorectal, and prostate disease progression and clinical outcomes differ considerably, yet a consistent theme emerges: cancer incidence and mortality are profoundly influenced by aging. Between 1991 and 2022, the overall cancer mortality rate in the United States decreased by 34%, resulting in roughly 4.5 million fewer deaths. This progress reflects advances in early detection, prevention, and treatment, as well as lifestyle changes, such as reduced smoking rates [1,4]. Despite these gains, the global cancer burden continues to rise, particularly among the elderly. Over 85% of all cancers occur in individuals older than 55, and the risk of developing invasive cancer after age 60 is more than twice that of younger populations [5]. Consequently, cancer is now the leading cause of death among adults aged 60–79, underscoring its intrinsic connection to biological aging and the progressive loss of cellular homeostasis [6].

Central to the initiation, progression, and response to treatment of cancer is the tumor microenvironment (TME), a highly dynamic and multifaceted ecosystem that surrounds and interacts with tumor cells [7]. Far from being a passive backdrop, the TME is composed of an array of cellular and non-cellular components, including immune cells, fibroblasts, endothelial and pericyte cells, adipocytes, extracellular matrix (ECM), cytokines, and blood vessels. These elements collectively generate a milieu that can alternately suppress or promote tumor growth, invasion, and eventual metastasis, depending on both local context and systemic factors such as age [8].

The TME is itself a product of evolution and adaptation, continuously reshaped by reciprocal interactions between tumor cells and their surrounding stroma. This ongoing evolution supports cancer cell survival, fosters immune evasion, and facilitates the dissemination of tumor cells to distant sites [9]. One of the defining features of the TME, particularly as cancer advances, is its immunosuppressive character. Initially, the immune system can recognize and eliminate nascent tumor cells, deploying cytotoxic T cells and natural killer (NK) cells as frontline effectors [10]. However, as the tumor adapts, it cultivates an immunosuppressive environment by recruiting regulatory T cells (Tregs), myeloid-derived suppressor cells (MDSCs), and tumor-associated macrophages (TAMs), all of which dampen effective anti-tumor immunity. In parallel, cancer cells often upregulate immune checkpoint molecules such as PD-L1, which inhibit T cell activity and further shield tumor cells from immune destruction. These processes not only enable unchecked tumor expansion but also diminish the effectiveness of immunotherapies; however, immune checkpoint inhibitors have provided new hope by targeting these pathways [11].

The structural components of the TME, particularly the ECM, play a critical role in defining tumor behavior. In healthy tissue, the ECM is crucial for maintaining cellular integrity, architecture, and signaling [12]. However, tumors manipulate ECM to their advantage, promoting excessive collagen deposition and matrix remodeling largely driven by cancer-associated fibroblasts (CAFs). This stiffened, fibrotic matrix increases tumor motility and invasion, thereby facilitating metastasis and contributing to therapy resistance [13]. The ECM further acts as a reservoir for a multitude of growth factors and cytokines, such as transforming growth factor beta (TGF-β) and vascular endothelial growth factor (VEGF), both of which drive cancer progression and support angiogenesis. Matrix metalloproteinases (MMPs), secreted by stromal and immune cells, degrade ECM components and pave the way for tumor cells to invade surrounding tissues and vasculature [14].

Angiogenesis, the formation of new blood vessels, is another signature hallmark of the TME, ensuring that tumors secure oxygen and nutrients necessary for their growth. The resulting tumor vasculature, however, is often leaky and disorganized, resulting in inefficient blood flow, persistent hypoxia, and nutrient gradients within the tumor mass [15]. Hypoxia itself drives further pathologic angiogenesis, upregulating VEGF and creating a vicious cycle in which tumor survival, aggressiveness, and therapy resistance are perpetuated [16]. CAFs, often the most abundant non-cancerous cell type in tumors, initially may suppress tumor growth but are eventually hijacked to promote proliferation, invasion, and resistance through the secretion of growth factors, cytokines, and matrix-modifying enzymes. By interacting with immune cells, CAFs reinforce local immunosuppression, thereby heightening the complexity of the microenvironment [17].

Metabolic reprogramming further distinguishes the TME. Tumor cells frequently adopt aerobic glycolysis (the Warburg effect), generating lactate and an acidic microenvironment that erodes immune cell function and promotes tumor survival [18]. Stromal cells, such as adipocytes, also contribute essential metabolites and lipids, fueling tumor cell growth and highlighting the metabolic codependence between cancer cells and their environment [19]. Ultimately, the TME preconditions potential future metastatic sites by secreting signaling molecules that foster the formation of a pre-metastatic niche, an environment in distant organs that is ideally suited for the colonization of circulating tumor cells [20].

Aging imposes profound changes on both the systemic immune system and the local tissue microenvironments, resulting in increased susceptibility to tumorigenesis, rapid progression, and metastasis [21]. Tissue aging is defined by increased cellular senescence, chronic low-grade inflammation (“inflammaging”), and persistent ECM remodeling [22]. These alterations collectively reshape the TME, generating more permissive and often more aggressively pro-metastatic conditions. With global life expectancy steadily increasing and projections suggesting that over 2 billion individuals worldwide will be over 60 by 2050, the clinical and societal imperative to understand age-associated TME changes has never been more urgent [23].

Recent advances have shed light on the intricate interplay between aging, immunity, and cancer. For decades, immunological aging research has focused predominantly on lymphocyte (especially T cell) intrinsic defects, declining efficacy, increased susceptibility to exhaustion, and impaired generation of new naive T cells by the aging thymus [24]. However, new perspectives underscore that age-associated deficiencies are not solely cell-intrinsic but result from broader microenvironmental deterioration, encompassing the bone marrow, thymus, secondary lymphoid organs, and the tumor itself [25].

Among the most groundbreaking recent findings is the identification of tumor-infiltrating, aging-associated dysfunctional CD8+ T cells (CD8+ T cells specifically associated with an aged TME: T_TAD_ cells). These cells, discovered in aged mouse models of cancer, are characterized by markers including PD-1 and TOX, as well as the presence of IL-7R, but lack typical exhaustion and memory markers such as TCF1, SLAMF6, and TIM3 [25]. In contrast, the T_TAD_ phenotype is best established in the preclinical context. Functionally, T_TAD_ cells demonstrate impaired cytotoxicity, lower granzyme B expression, higher TNF production, and overall poor tumor control capabilities. Strikingly, when young, vigorous CD8+ T cells from healthy animals are introduced into the aged TME, they too adopt this dysfunctional T_TAD_ phenotype, highlighting the central role of the aged TME, rather than solely T cell age, in driving immune effector failure [26]. To clearly differentiate mouse and human data, this distinction emphasizes that the T_TAD_ phenotype is well-supported in aged mouse tumors, while human data are still preliminary and mainly correlative.

The presence and expansion of T_TAD_ cells in the aged TME reflect broader failures in intercellular communication essential for effective tumor control. With age, crosstalk between NK cells, type 1 conventional dendritic cells (cDC1s), and CD8+ T cells is weakened, resulting in defective antigen presentation and T cell priming [26]. Reductions in cDC1s and NK cells within the aged TME further compromise immune surveillance. These defects extend into secondary lymphoid organs, which, as they age, lose the ability to sustain naive T cells and coordinate robust anti-tumor responses [27]. Although T_TAD_ cells may partly explain why older hosts exhibit weakened responses to immunotherapy in preclinical studies, there is currently limited direct evidence that T_TAD_-like cells significantly impact human cancer outcomes. Larger, age-specific human cohorts, combined with functional and longitudinal data, are essential to accurately assess their true prevalence and prognostic importance.

The growth of immunosuppressive populations, such as Tregs and MDSCs, is paralleled by a diminished effector population, including cytotoxic CD8+ T cells, NK cells, and cDC1s, which together tip the balance away from tumor control [28]. Chronic inflammation, largely driven by accrual of senescent cells with their pro-inflammatory senescence-associated secretory phenotype (SASP), further degrades the local tissue environment and instigates ECM remodeling. In contrast, increased fibrosis driven by CAF activity encourages cancer cell invasion and therapy resistance. These changes not only foster metastasis but also blunt the effectiveness of existing treatments, explaining why older cancer patients often fare worse [29].

Metabolic reprogramming within the aged TME adds a further layer of complexity, as tumor and stromal cells adapt to aging-induced nutrient and oxygen deprivation by shifting towards glycolytic and other alternative metabolic pathways, supporting sustained tumor outgrowth despite environmental constraints [29]. Therapeutically, recognizing the unique biology of the aged TME is critical. Standard therapies often fail or are less effective in elderly patients due to these age-related changes [30]. Novel strategies are under development to target the aged TME, specifically senolytics to clear senescent cells, immune-modulating therapies to restore effective antitumor responses, agents targeting the ECM and CAF interactions, and interventions aimed at reactivating efficient antigen presentation and T cell priming (such as anti-CD40 therapy). However, each of these strategies faces significant challenges given the complex, adaptive, and resilient nature of the aged TME [31].

In this review, we summarize current understanding of how aging remodels the TME, emphasizing the interaction between extrinsic factors (environment- and stroma-driven) and intrinsic mechanisms (cellular aging) that lead to immune dysfunction.

Of particular interest is the potential to target T_TAD_ cells or reverse their development, offering new strategies to improve outcomes for elderly cancer patients. Aging clearly reprograms the TME, creating a pro-metastatic environment characterized by cellular senescence, chronic inflammation, ECM dysregulation, and immune decline. Addressing these age-related changes is crucial to reduce the growing cancer burden in aging populations. Innovative, age-specific therapeutic approaches, guided by integrated insights from immunology, cancer biology, and gerontology, are likely to produce meaningful translational progress for elderly patients.

Briefly, we conducted a structured search of PubMed, Scopus, and Web of Science for articles published between January 2020 and October 2025. We used combinations of keywords related to aging, tumor microenvironment, ECM remodeling, immunosenescence, and cancer progression. Studies were selected based on relevance to mechanistic insights, aging-specific biology, and contributions to tumor progression.

## 2. Aging Hallmarks in the Tumor Microenvironment

In the aged TME, metastatic progression is driven by a complex interplay of molecular pathways stemming from age-related cellular and tissue dysfunction [32,33,34]. Aging leads to the buildup of senescent cells, which, although initially tumor-suppressive, shift toward a pro-tumorigenic phenotype through the sustained release of the SASP (senescence-associated secretory phenotype). SASP includes a variety of cytokines (e.g., IL-6, IL-8), chemokines (CXCL1, CCL2), growth factors (VEGF, TGF-β), and proteases, such as MMPs, that collectively modify the TME, promote tumor invasion, and support metastatic spread [35]. A key element in this process is the activation of vital signaling pathways, such as TGF-β, MAPK/AKT, and STAT3, which are triggered by SASP factors and significantly promote EMT, cellular plasticity, and migratory behaviors [36]. TGF-β facilitates EMT, enabling tumor cells to detach, invade, and survive in the circulation, while SASP-driven MMP1, MMP3, and other matrix metalloproteinases degrade the ECM (extracellular matrix), creating physical pathways for tumor dissemination [37]. Additionally, chronic inflammation and ongoing oxidative stress within aging tissues accelerate DNA damage and telomere shortening, fueling genetic instability and enhancing metastatic potential [38].

The aged ECM undergoes substantial alterations due to LOX-mediated collagen cross-linking, as well as the accumulation of fibronectin and hyaluronan. These changes stiffen the matrix, activate the integrin-FAK and YAP/TAZ mechanotransduction pathways, and foster an environment optimized for cancer cell migration and invasion [32]. CAFs under SASP influence perpetuate fibrosis and promote ECM remodeling, while metabolic reprogramming (enhanced glycolysis and OXPHOS) generates metabolites like lactate and ROS, further supporting tumor progression. Immune dysfunction is another hallmark; senescent cells and aged stroma secrete factors that recruit immunosuppressive MDSCs and skew TAMs toward a pro-tumoral M2-like phenotype [17]. This immunosuppressive shift diminishes cytotoxic T cell activity and impairs natural killer cell function, allowing tumor cells to escape immune surveillance. SASP-induced expression of PD-L1 by both stromal and tumor cells further cripples anti-tumor responses [33].

Angiogenesis within the aged TME is characterized by abnormal, leaky vasculature driven by increased VEGF secretion. In aged tissues, VEGF-A expression increases by approximately 2–4 times, but functional vessel density decreases by 20–40%, resulting in leaky, poorly perfused vasculature with about 30–50% higher permeability and oxygen tensions falling below roughly 15 mmHg. This vascular dysfunction leads to chronic hypoxia, which selects for aggressive, therapy-resistant clones and supports further EMT-mediated metastatic dissemination. Hypoxia also intensifies metabolic and epigenetic reprogramming, creating stress-adapted cancer cell populations capable of thriving under adverse conditions [34,35].

**Figure 1:**
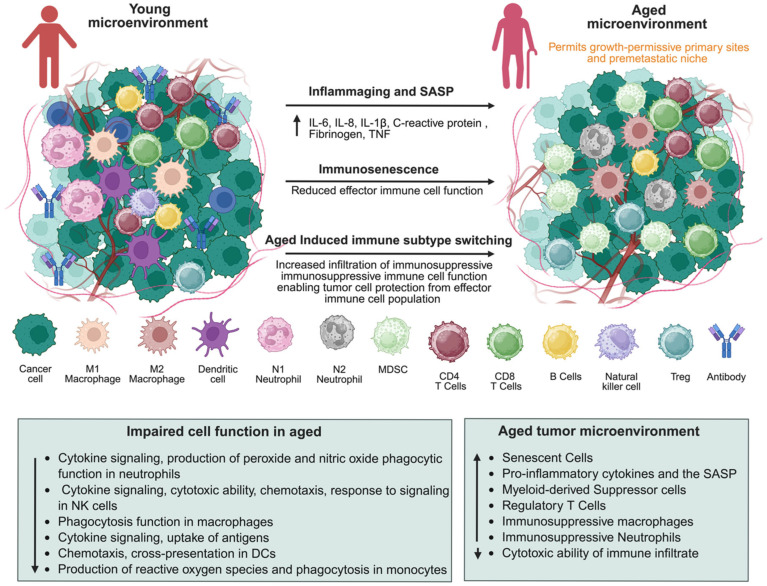
**Age-associated alterations in the tumor microenvironment (TME) promote metastasis.** Compared with the young TME, the aged TME exhibits inflammaging, immunosenescence, and immune subtype switching toward immunosuppressive phenotypes (MDSCs, M2 macrophages, Tregs, N2 neutrophils). These changes, driven by elevated SASP cytokines (IL-6, IL-8, IL-1β, TNF, etc.), impair the function of effector immune cells and establish a growth-permissive, pre-metastatic niche. Lower panels summarize impaired immune functions and hallmark features of the aged TME that collectively facilitate tumor progression in the elderly host. ↑ Indicates an increase; ↓ indicates a decrease in the cell population and functions. Created in BioRender. Kumar, S. (2025) https://BioRender.com/i37v6eo.

Telomere attrition coupled with sustained oxidative stress-induced DNA damage diminishes cellular replicative potential, driving senescence and establishing a mutagenic, tumor-permissive niche. In parallel, epigenetic reprogramming, facilitating immune evasion and metastatic progression, is characterized by aberrant DNA methylation patterns and histone modifications. In sum, the aged tumor microenvironment is distinguished by enhanced SASP activity, chronic inflammation, ECM remodeling, immune suppression, aberrant angiogenesis, and metabolic dysregulation. These factors orchestrate a landscape where molecular signals and structural changes collectively drive tumor metastasis, emphasizing the need for therapies that target the crosstalk between aging biology and cancer progression [36]. This is comprehensively summarized in Figure 1.

## 3. Molecular Mechanisms and Signaling Pathways

### 3.1. Extracellular Matrix (ECM) Remodeling

The central mechanistic event in age-related tumor progression is ECM remodeling, which involves biochemical and biomechanical changes that create an environment that supports cancer growth and metastasis [37]. In an aging microenvironment, dystrophic regulation of enzymes in modifying the ECM, such as the matrix metalloproteinases (MMPs) and lysyl oxidases (LOXs), mediates an increase in the rates of degrading structural components such as collagen and elastin, with the upregulation of abnormal crosslinking [38,39]. Overactivation of the MMPs degrades the basement membrane, promoting tumor cell invasion, while collagen crosslinking by LOX stiffens the ECM. Elevated stiffness is a prominent feature of cancer and aging. For example, in aged tissues, stiffness increases from approximately 1–2 kPa to 3–5 kPa, and tumors can reach levels of 10–20 kPa, and the stiffness magnitude varies in different tumor conditions, which are sufficient to trigger integrin clustering and FAK/Src activation—a biomechanical signal that enhances integrin clustering and focal adhesion formation or YAP/TAZ signaling pathways [40,41,42]. All these alterations together modulate the tissue architecture, decrease tissue elasticity, and create conditions suitable for initiating and propagating tumors [43,44,45]. The modification in ECM stiffness and composition has far-reaching consequences for mechano-transduction paths, which affect the behavior of tumor cells [42]. Increased rigidity enhances the integrin-driven activation of focal adhesion kinase (FAK) and Src family kinases, leading to the subsequent activation of Rho GTPases, notably RhoA, Rac1, and Cdc42 [46,47]. These signaling processes coordinate cytoskeletal motion, cell polarity, and migration, thereby helping cancer cells acquire invasive phenotypes [45]. Moreover, the coverage of integrin signaling by the MAPK and PI3K/Akt pathways contributes to the processes of proliferation and survival, as well as resistance to apoptosis, which further exacerbates the possibility of therapeutic regulation in old tissues [48]. A major gap in the contemporary understanding is the distinction between the age effect, ECM remodeling, and tumor-induced ECM changes, as all share common molecular mediators. Both aging and tumor development alter the extracellular matrix, but they do so through different initiating mechanisms and timelines. Aging involves gradual collagen cross-linking, elastin degradation, and reduced matrix turnover, primarily due to chronic oxidative stress and senescence signals such as LOX upregulation and decreased MMP activity. Conversely, tumor-driven remodeling is reactive and localized, characterized by excessive MMP activity, abnormal ECM deposition by cancer-associated fibroblasts (CAFs), and persistent integrin/FAK mechanotransduction that promotes invasion. Although mediators like LOX, MMPs, and TGF-β are involved in both, their regulation differs: aging leads to a permissive, fibrotic ECM through slow systemic stiffening, while tumorigenesis exploits these changes to accelerate metastatic growth. Recognizing these differences is crucial for identifying biomarkers that differentiate age-related structural alterations from tumor-specific ECM reprogramming, ultimately improving targeted therapies for aged tissues. This degree of overlap hinders the development of accurate biomarkers to distinguish between pathologic remodeling and physiologic aging [49]. In addition, although integrin and Rho GTPase pathways have established roles in regulating the ECM, their activation patterns in the aged tumor microenvironment are not well-defined spatiotemporally, which constrains ECM regulation by targeted interventions [50].

The best way to rectify such gaps is the integrative application of multi-omics and the use of advanced imaging and temporal studies focusing on real-time ECM remodeling [51]. Direct inhibition of MMPs and LOXs has the disadvantages of both systemic toxicity and the inability to target a specific MMP; therefore, one potential solution may be upstream regulation of a signaling node, such as FAK, or the expression of signaling-specific subtypes of integrins [52,53,54,55]. Novel approaches, such as engineered peptides that interfere with integrin-ECM interactions or nanocarriers delivering siRNA targeting isoforms of LOX, are promising tools for selectively normalizing ECM stiffness without affecting physiological tissue repair. Furthermore, given the recent development of biomimetic in vitro models capable of recapitulating age-related changes in the ECM, age-specific therapeutics can be identified whose efficacy in the unique biomechanical and biochemical environment of aged tissues can be determined more quickly [46]. Filling the gap between mechanistic knowledge and regulatory manipulation may help turn ECM remodeling, which is currently a risk factor in promoting tumors, into a therapeutic opportunity, especially among cancer patients who are elderly.

### 3.2. Inflammation and Cytokine Signaling

**Figure 2:**
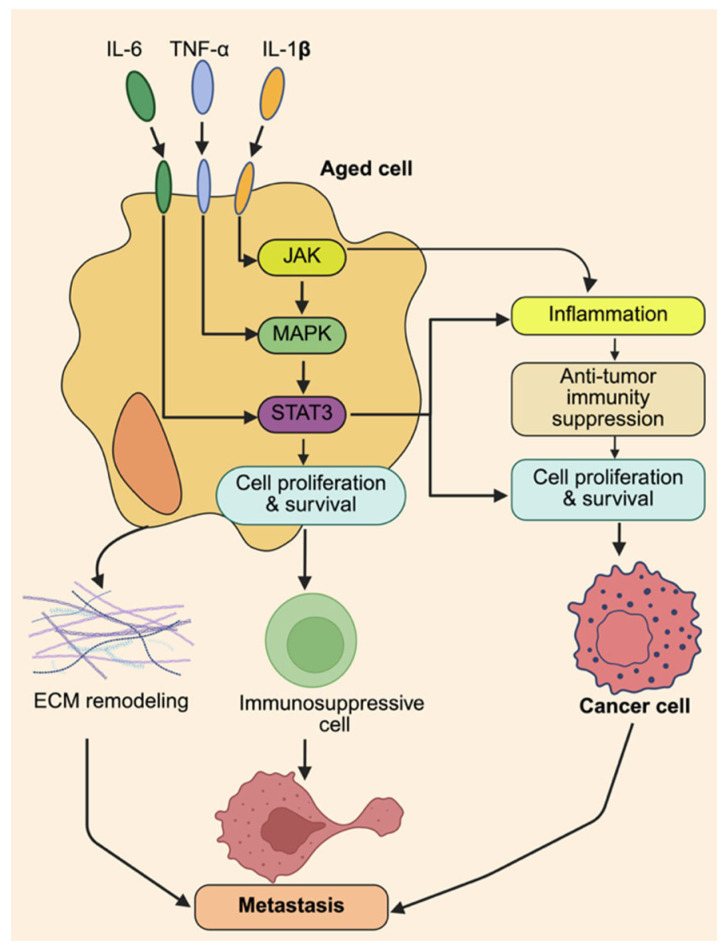
**Molecular signaling mechanisms driving metastatic progression within the aged TME.** This schematic illustrates how aging-related cytokines and signaling pathways collaborate to facilitate tumor metastasis. In the aged microenvironment, increased levels of IL-6, TNF-α, and IL-1β activate their receptors on tumor and stromal cells, leading to downstream signaling through JAK, MAPK, and STAT3 pathways. These pathways activate transcription programs that boost cell proliferation and survival, promote inflammation, and weaken anti-tumor immune responses. Continuous activation of STAT3 and NF-κB encourages angiogenesis and the recruitment of immunosuppressive cells. Additionally, persistent cytokine signaling drives extracellular matrix remodeling, creating a favorable environment for tumor invasion. Overall, these interconnected molecular processes support metastatic spread within the aged tumor microenvironment. Created in BioRender. Kumar, S. (2025) https://BioRender.com/i37v6eo.

Chronic low-grade inflammation, sometimes called inflammaging, present in the TME creates an ideal environment that promotes cancer progression and metastasis [6,51]. Elevated levels of pro-inflammatory cytokines, including interleukin-6 (IL-6), tumor necrosis factor-alpha (TNF-α), and interleukin-1 beta (IL-1β), are characteristic of aging tissues (Figure 2) [56]. These cytokines exert both autocrine and paracrine effects, recreating the TME by boosting tumor cell survival, motility, and invasion. IL-6 activates epithelial–mesenchymal transition (EMT) and encourages metastatic growth [57]. TNF-α promotes the expression of adhesion proteins, facilitating the intravasation and extravasation of cancer cells (Figure 1). IL-1β stimulates the production of matrix-degrading enzymes, enabling tissue invasion. Together, these mediators form a self-amplifying inflammatory cycle that becomes intensified and prolonged in older individuals, in whom immune-resolving processes often become compromised. At the signal transduction level, these cytokines activate pro-tumorigenic pathways such as NF-κB, STAT3, and MAPK, aligning them with extracellular inflammatory signatures and tumor-promoting transcriptional programs [49]. NF-κB activation by TNF-α and IL-1β enhances gene expression related to cell survival, angiogenesis, and immune suppression. IL-6 mainly signals through the JAK/STAT3 pathway, which promotes proliferation, stemness, and metastatic potential [50]. Activation of MAPK pathways links inflammation with cytoskeletal remodeling, increasing cellular migratory capacity and enabling adaptation to microenvironmental changes. These pathways often work together, creating redundancy that complicates therapeutic targeting in the aged TME. Chronic cytokine signaling is particularly harmful because it can induce angiogenesis while simultaneously suppressing anti-tumor immunity (Figure 2) [58]. Persistent NF-κB and STAT3 activation increases VEGF production [54], fostering the growth of abnormal, leaky blood vessels that supply nutrients and support metastasis. Concurrently, these pathways facilitate the recruitment of MDS and regulatory T cells, which inhibit cytotoxic T lymphocyte activity [55], further tipping the immune balance toward evasion. This combination makes chronic inflammation during aging a highly effective tumor promoter. A major research challenge is distinguishing between inflammation caused by normal aging and that driven by tumors, as they involve overlapping cytokine networks. This overlap limits the development of interventions that selectively suppress pathological inflammation without impairing essential immune surveillance. Systemic anti-inflammatory treatments also carry risks of poor specificity and weakened host defenses. Potential therapies include high-resolution immunomodulation targeting upstream regulators like IL-6R or TNF-α with monoclonal antibodies, combined with downstream inhibitors such as STAT3 or NF-κB small-molecule drugs [59]. Another promising approach is using senolytic agents to eliminate senescent cells in the aged TME, which produce inflammatory cytokines and raise baseline inflammation levels [60]. Combining these precision strategies with biomarker-guided patient selection may allow us to break the link between inflammation and metastasis while preserving critical immune functions.

### 3.3. Senescence-Associated Secretory Phenotype (SASP)

**Figure 3:**
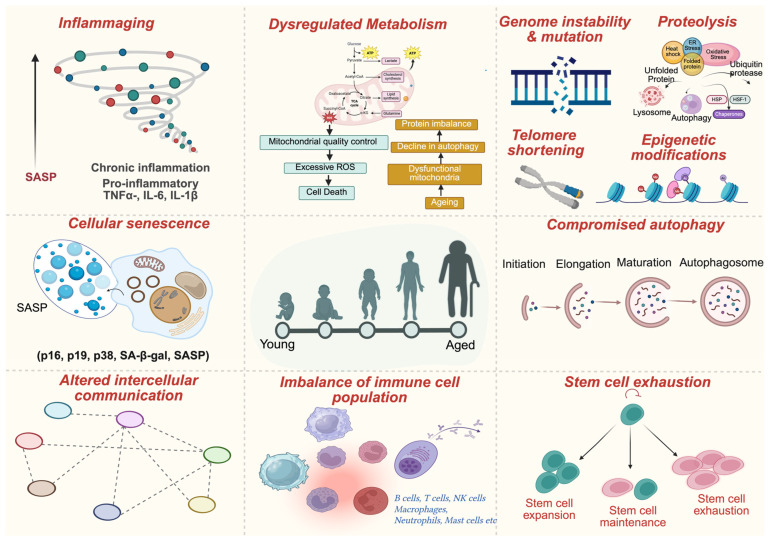
**Cellular and molecular hallmarks of aging contributing to tumor-promoting microenvironments.** Aging is characterized by interconnected alterations at the cellular level, including stem cell exhaustion, cellular senescence, and disrupted intercellular communication, and at the molecular level, including telomere attrition, genomic instability, epigenetic alterations, loss of proteostasis, mitochondrial dysfunction, and impaired autophagy. These changes collectively compromise tissue homeostasis and create environments that are permissive to tumor initiation and progression. Created in BioRender. Kumar, S. (2025) https://BioRender.com/i37v6eo.

SASP is a phenotype of senescence in aging tissues, where senescent cells release a persistent stream of bioactive molecules and cease dividing, yet retain their viability and metabolism [61]. SASP transforms the cancer-inhibitory property of senescence into tumorigenicity in the aging TME by reshaping tissues, facilitating constant inflammatory conditions, and creating environments conducive to metastasis. SASP is highly heterogeneous and often contains pro-inflammatory cytokines (IL-6, IL-8), growth factors (VEGF, HGF), and proteases, MMPs (Figure 3) [33]. These influences interact and can modify the extracellular matrix, induce angiogenesis, and increase the motility and invasiveness of tumor cells, thereby promoting metastatic colonization in other organs. The pro-metastatic role of SASP is linked to its properties, which alter intracellular interactions within the TME. Cytokines and growth factors secreted by senescent fibroblasts may reprogram surrounding epithelial cells via epithelial-to-mesenchymal transition [62], a key step in invasion and spreading. Proteases that degrade basement membranes enhance the physical mobility of cancer cells. Additionally, chemokines released by SASP-positive cells further stimulate anti-tumor immune responses by attracting immunosuppressive cells, such as MDSCs and regulatory T cells, which are drawn to the chemokines produced by SASP-positive cells [63]. These effects are amplified in aged tissues, where the continued presence of senescent cells increases as immune clearance mechanisms decline with age, while the activity of SASP remains unaffected by the decreased capacity to clear cells. SASP is generated at the molecular level, tightly regulated by signaling networks associated with the DNA damage response (DDR) and the metabolic state. Persistent activation of the DDR due to telomere attrition or genotoxic stress initiates a p53/p16INK4a-induced growth arrest and stimulates NF-κB and C/EBPβ transcription factors that activate SASP genes [64]. The translation of SASP components is controlled by mTOR signaling, a key regulator of cell metabolism that sustains SASP secretion. These pathways form a positive-feedback loop, continually activating SASP factors that increase DNA damage and stress, thus reinforcing the senescent state and promoting their proliferative effects on cancer. One challenge is that SASP can be harmful in chronic aging but also has positive roles in wound healing and tissue regeneration; therefore, indiscriminate reduction can be problematic. Current treatments, including broad-spectrum anti-inflammatory agents or mTOR inhibitors (e.g., rapamycin), risk disrupting essential physiological processes [65]. Emerging targeted approaches focus on SASP drivers. Senolytic drugs aim to eliminate only senescent cells, while senomorphic agents modify secretion without causing cell death. Temporally controlled therapy using SASP suppression only during periods of metastatic risk is another promising strategy. Future single-cell transcriptomics and proteomics could help identify patient-specific SASP signatures, enabling interventions that limit the tumor-promoting activity of SASP while preserving its regenerative functions. Such selective manipulation of SASP could be key to reconstructing the aging TME into a less conducive environment for cancer metastasis [66].

### 3.4. Immune Cell Dysfunction

The dysfunction of immune cells in the aging TME is a key factor in metastasis, influenced by both quantitative and qualitative changes in immune cell numbers [67]. The decreased production of naive T cells that occurs with immunosenescence, as the thymus involutes, shifts the T cell repertoire towards memory T cell phenotypes with reduced proliferation. In response, cytotoxicity of NK cells [68] declines, and dendritic cells become less effective at antigen presentation. These deficiencies impair immune surveillance, allowing circulating tumor cells to spread and form metastatic colonies. Chronic inflammation, driven by aging, also promotes the recruitment of immunosuppressive cells, such as MDSCs and Tregs, to establish immunosuppressive environments that inadvertently support tumor growth while promoting immune tolerance over cancer progression. Immune suppression in the aged TME is further characterized by the overexpression of checkpoint molecules, notably PD-1 on T cells and PD-L1 on tumor and stromal cells, as well as CTLA-4 on regulatory cells [69]. When transduced, PD-1 or CTLA-4 sends inhibitory signals to dampen TCR activity, reducing cytokine production, cell proliferation, and cytolytic function. This occurs through downstream inhibition, including SHP2-dependent dephosphorylation of proximal TCR signaling molecules and the suppression of PI3K/Akt activation [70,71]. Meanwhile, tumor cells exploit these inhibitory pathways to create a tolerogenic niche that shields them from immune attacks. In the elderly, chronic antigen stimulation and impaired co-stimulatory signaling lead to increased checkpoint-mediated exhaustion, which diminishes the effectiveness of anti-tumor responses to conventional activation signals.

The defect in adaptive immunity is not the only issue; poor TCR signaling is also reflected in impairments of NK cells and macrophages, which have their own inhibitory receptor systems. Integrating molecules with metabolic checkpoints, such as TIGIT, LAG-3, and TIM-3, promotes a hypo-responsive state of the immune system [72]. Through this complex suppression, circulating metastatic cells can survive in circulation as well as thrive in secondary organs, where secondary immunologic escape mechanisms are similar to those of the primary tumor. Significant limitations to existing therapeutic strategies lie in the fact that immune checkpoint blockade, which is successful in subsets of younger patients, fails to exert its effect in the older population, in part due to the prior depletion and dysfunction of effector immune cell populations. Solving this constraint may require combinatorial approaches to reverse the immune cell fitness, in addition to checkpoint inhibition. This may include cytokine therapy to enhance T cell and NK cell activity, metabolic reprogramming to overcome exhaustion, or adoptive cell therapy that is tailored to aged immunity [73,74]. Single-cell immune profiling of cancer patients with advanced age should help detect aberrant subsets and signaling pathways that lead to unchecked inhibitory brakes, followed by the precision and reinvigoration of anti-tumor immune responses. Correction of these interrelated defects could normalize effective immune surveillance and minimize the burden of metastases during the aging process in the population [75].

### 3.5. Metabolic Reprogramming

The alteration of metabolism in an aging TME significantly impacts tumor development and metastatic spread [76]. Aging tissues can cause changes in nutrient supplies, oxygen levels, and metabolite accumulation, creating a microenvironment that favors cancer cell populations with high metabolic flexibility [77]. The tumor’s ability to switch between glycolysis and oxidative phosphorylation, depending on nutrient availability, offers a selective advantage to tumor cells growing in aged hosts that face changing environments and energy challenges. This metabolic flexibility supports proliferation and meets the biosynthetic and redox requirements for invasion and metastasis [78]. In aged tissues, a subset of stromal cells, specifically cancer-associated fibroblasts, can contribute to this process through metabolic reprogramming, primarily shifting toward a glycolytic phenotype and releasing lactate, pyruvate, and other metabolic products that tumor cells can then utilize through metabolic coupling. Nutrient availability in the aged TME changes markedly, along with disruptions in key metabolic signaling pathways such as mTOR and AMPK [79]. Age-related chronic inflammation and growth factor signaling can increase mTOR activity, promoting the synthesis of proteins, lipids, and nucleotides, and facilitating metastatic colonization. Conversely, AMPK, the primary energy sensor activated during nutrient stress, is often suppressed during aging due to mitochondrial alterations and persistent nutrient signaling, weakening the tumor-suppressing functions of autophagy and oxidative stress. This imbalance allows continued anabolic activity in nutrient-poor metastatic environments, supporting tumor cell survival and plasticity. Hypoxia, which occurs more frequently in aged tissues due to vascular dysfunction, further promotes metabolic reprogramming by stabilizing hypoxia-inducible factors (HIFs). These factors upregulate glycolytic enzymes, angiogenic factors, and genes associated with EMT [80], linking metabolism to invasive capabilities. HIF-regulated mechanisms work in concert with mTOR to sustain metastatic potential even under low-oxygen conditions. Additionally, competition for nutrients between tumor and immune cells in the elderly TME further impairs T cell metabolism, contributing to immune suppression and increased metastasis. A critical area of investigation is the limited understanding of how age-related metabolic changes in stromal and immune compartments influence tumor cell metabolism at metastatic sites. Current treatments targeting cancer metabolism often overlook the unique metabolic shifts associated with aging, resulting in suboptimal results. Therefore, the metabolic interactions within aged TMEs need comprehensive profiling to uncover vulnerabilities specific to elderly patients. Proposed approaches include dual-targeting therapy, which involves blocking mTOR signaling while activating AMPK to restore metabolic balance, as well as methods to enhance nutrient exchange between stromal and tumor cells, thereby returning these processes to normal levels [81]. Dietary interventions and metabolic adjuvants could enhance anti-tumor immune responses and control metastasis by modulating the metabolic crosstalk between immune and cancer cells. In theory, reprogramming the aging metabolic environment into a less supportive niche could reduce tumor dissemination, thereby lowering risks in older populations [82].

## 4. Discussion of Major Age-Related Cancers

### 4.1. Breast Cancer in the Aging Population

Breast cancer demonstrates a striking age-dependent incidence pattern, with approximately 80% of cases occurring in women over 50 years of age [83]. The biological behavior of breast tumors in elderly patients differs significantly from that in younger individuals, mainly due to age-related changes in the TME. As women age, their breast tissue undergoes involution, a process characterized by the replacement of glandular elements with adipose tissue and increased stromal fibrosis [84]. This structural remodeling creates a permissive niche for cancer development through several mechanisms. The accumulation of senescent cells in aging breast tissue contributes to a chronic inflammatory state through the SASP (Senescence-Associated Secretory Phenotype), which releases pro-inflammatory cytokines such as IL-6 and IL-8, matrix MMPs, and growth factors that promote tumor initiation and progression [85]. Importantly, the aged mammary stroma exhibits increased stiffness due to enhanced collagen cross-linking by enzymes like LOX, creating mechanical cues that activate pro-tumorigenic signaling pathways in epithelial cells, including the YAP/TAZ and TGF-β pathways. Immunologically, aging leads to thymic involution and a decrease in the production of naive T cells, resulting in a less diverse T-cell repertoire and impaired immune surveillance [86]. An increase in immunosuppressive cell populations, such as Tregs and MDSCs, within the TME compounds this immunosenescence, as summarized in Table 1, with other cancers. Clinically, breast tumors in older women are more likely to be hormone receptor-positive and HER2-negative, which generally portends a better prognosis but also presents unique therapeutic challenges. While endocrine therapies remain highly effective, the management of elderly breast cancer patients must carefully balance treatment efficacy with potential toxicities, particularly in those with comorbidities [87]. Emerging strategies targeting age-specific aspects of the TME, including senolytic therapies to eliminate pro-tumorigenic senescent cells and immune modulation approaches to overcome age-related immune dysfunction, hold promise for improving outcomes in this growing patient population [88].

### 4.2. Prostate Cancer and Aging

Prostate cancer exemplifies an age-related malignancy, with incidence rates rising sharply after age 50 and most cases diagnosed in men over 65. The strong link between aging and prostate cancer reflects significant changes in the prostate microenvironment that occur with age [89]. The aging prostate undergoes notable architectural changes, including progressive glandular atrophy, increased stromal fibrosis, and chronic inflammation, collectively referred to as proliferative inflammatory atrophy. This inflammatory environment, driven by age-related oxidative stress and repeated microtrauma, creates favorable conditions for malignant transformation through various mechanisms [90]. Chronic inflammation leads to the sustained activation of NF-κB signaling, which promotes cell survival and growth while also fostering an immunosuppressive environment by recruiting regulatory T cells and tumor-associated macrophages. Androgen signaling, which is vital in prostate cancer, shifts considerably with age. Although circulating testosterone levels decline, intraprostatic androgen metabolism becomes irregular, potentially aiding the development of castration-resistant prostate cancer (CRPC) [54]. The aged prostate stroma also undergoes functional changes, with senescent fibroblasts adopting a CAF-like phenotype that actively supports tumor growth by secreting growth factors and enzymes that modify the extracellular matrix. Clinically, prostate cancer in older men often appears less aggressive but may also develop increased resistance to therapy [91]. Current treatment options need to carefully balance oncologic control with quality-of-life considerations for older patients. New therapeutic strategies targeting features of the aging tumor microenvironment, including anti-inflammatory treatments and therapies aimed at senescent cells, are actively under investigation to improve outcomes for elderly prostate cancer patients [92].

### 4.3. Lung Cancer in the Elderly

Lung cancer incidence peaks in the seventh and eighth decades of life, making it primarily a disease of older adults. The aging process significantly impacts the lung microenvironment, creating conditions that promote both the initiation and progression of pulmonary malignancies [6]. Chronic exposure to environmental carcinogens, especially tobacco smoke, works synergistically with age-related changes to drive lung cancer development. The aged lung exhibits multiple structural changes, including reduced elastic recoil, alveolar enlargement, and increasing fibrosis, which contribute to the formation of a pro-tumorigenic environment [93]. A key characteristic of the aging lung is the accumulation of senescent cells in both the epithelial and stromal areas, which, through their SASP, induce a persistent inflammatory state that fosters genomic instability and epithelial–mesenchymal transition [94]. Immunologically, the aged lung undergoes notable shifts in immune cell populations and function, marked by a decline in naive T cells and an increase in exhausted or senescent T cells, which impairs antitumor immune responses [95]. This immunosenescence is especially relevant in the context of immunotherapy, which has transformed lung cancer treatment but tends to be less effective in older patients. The aged lung also shows altered metabolic profiles, with heightened glycolysis and lactate production contributing to an immunosuppressive tumor microenvironment [87]. Clinically, elderly lung cancer patients often present with more advanced disease and have historically been underrepresented in clinical trials, creating uncertainties about optimal treatment. While targeted therapies for oncogene-driven tumors (such as EGFR or ALK inhibitors) remain effective regardless of age, the benefits of traditional chemotherapy and immunotherapy may be diminished in older individuals [96]. Current research is focused on developing age-specific treatment strategies and biomarkers better to predict therapeutic responses in elderly patients with lung cancer.

### 4.4. Bladder Cancer and Age-Related Changes

Bladder cancer exhibits one of the strongest age-dependent incidence patterns among solid tumors, with a median age at diagnosis of 73 years. The aging urinary tract undergoes multiple structural and functional changes that contribute to bladder carcinogenesis [97]. The urothelium in elderly individuals shows increased permeability, reduced regenerative capacity, and accumulation of DNA damage, all of which cause a predisposition to malignant transformation [98]. A hallmark of the aged bladder microenvironment is chronic low-grade inflammation, driven by factors such as recurrent urinary tract infections, urinary stasis, and cellular senescence. This persistent inflammatory state leads to the sustained activation of NF-κB and STAT3 signaling pathways, which promote cell survival and proliferation while creating an immunosuppressive environment [38]. The aged bladder stroma becomes increasingly fibrotic, with excessive deposition of collagen and other extracellular matrix components that facilitate tumor invasion and metastasis [99]. Senescent fibroblasts in the bladder wall acquire a pro-inflammatory phenotype and secrete matrix-remodeling enzymes that degrade the basement membrane, promoting the dissemination of cancer cells. Immunologically, aging leads to profound changes in the bladder’s immune landscape, characterized by decreased effector T cell function and increased infiltration of immunosuppressive cell populations, including MDSCs and Tregs [77]. These age-related immune changes have significant implications for immunotherapy response, particularly for Bacillus Calmette–Guérin (BCG) therapy in non-muscle-invasive bladder cancer, which shows reduced effectiveness in elderly patients [100]. Clinically, bladder cancer in older adults often presents at more advanced stages and exhibits more aggressive biological behavior. Treatment decisions must carefully balance oncologic control with quality of life, particularly in frail elderly patients. Emerging therapeutic strategies targeting age-specific features of the bladder TME, including senolytic therapies and immune modulation approaches, may help improve outcomes for elderly bladder cancer patients [101].

### 4.5. Colorectal Cancer in the Aging Population

Colorectal cancer (CRC) incidence increases dramatically with age, with over 90% of cases occurring in individuals over 50 years of age [102]. The aging gastrointestinal tract undergoes profound changes that create a permissive microenvironment for the development of colorectal carcinogenesis. A central feature of age-related changes in the colon is the alteration of the gut microbiome, known as dysbiosis, characterized by a decrease in beneficial butyrate-producing bacteria and an increase in pro-inflammatory species [39]. This microbial shift contributes to a chronic inflammatory state in the colonic mucosa, leading to epithelial damage and potential malignant transformation. The aged colonic epithelium exhibits reduced regenerative capacity and increased genomic instability, while the underlying stroma becomes progressively fibrotic due to activation of CAFs and excess extracellular matrix deposition [103]. Cellular senescence plays a dual role in the aging colon; senescent epithelial cells accumulate DNA damage, while senescent stromal cells create a pro-tumorigenic niche through their SASP [33]. The aged colonic immune system undergoes significant remodeling, characterized by a decrease in mucosal immunity and an increase in the infiltration of immunosuppressive cell populations, which facilitates immune evasion by tumors. Vascular changes in the aging colon, including increased permeability and altered angiogenesis, further contribute to tumor progression and metastatic spread [39]. Clinically, colorectal cancer in elderly patients often shows distinct molecular features, including higher rates of microsatellite instability and BRAF mutations, which have important therapeutic implications. While surgical resection remains the primary curative treatment, older patients often face challenges related to treatment tolerance and postoperative recovery [104]. Systemic therapies must be carefully tailored to account for age-related changes in drug metabolism and increased susceptibility to toxicities. Emerging strategies targeting age-specific aspects of CRC biology, such as microbiome modulation and senolytic therapies, offer promising avenues for improving outcomes in elderly CRC patients. The development of comprehensive geriatric assessment tools has become increasingly important for guiding treatment decisions in this vulnerable population [105].

## 5. Conclusions and Perspective

The aging TME is increasingly recognized as a key factor in driving metastatic cancer progression, with age-related changes including immune dysfunction, stromal modifications, ECM remodeling, and vascular decline. Together, immunosenescence, inflammaging, and pro-metastatic secretory profiles create a niche that encourages tumor cell spread and therapy resistance in older adults. Despite these insights, our mechanistic understanding remains limited, primarily due to the reliance on young preclinical models and age-mismatched in vitro systems that fail to capture the complexity of human aging. Advancing this field requires the use of age-faithful models, such as geriatric mice, age-matched organoids, and primary stromal and immune cells from elderly donors, combined with high-resolution single-cell and spatial multi-omics to unravel the cellular heterogeneity of the aged TME. Identifying vulnerabilities specific to aging tissues could lead to the development of targeted, age-adapted therapies. As cancer rates increase among aging populations, focusing research on the aged TME is critical for improving outcomes and reducing metastatic spread in elderly patients.

### Limitations of the Study

This review is limited by the reliance on preclinical studies using young or age-mismatched models, small sample sizes, and species-specific differences. Moreover, some mechanistic insights are derived from in vitro systems that may not fully replicate the tumor microenvironment, potentially affecting the generalizability of findings to human aging and cancer. Despite significant clinical advances in cancer immunotherapy, its effectiveness in older adults remains limited by age-related declines in immune function. Immunosenescence is characterized by a reduction in the production of naïve T cells, decreased TCR diversity, and the accumulation of exhausted or senescent T cells, which leads to weaker responses to immune checkpoint inhibitors and related treatments. The aged tumor microenvironment adds further complexity, characterized by increased infiltration of regulatory T cells (Tregs), myeloid-derived suppressor cells (MDSCs), and higher levels of pro-inflammatory cytokines, all of which promote immunosuppression and hinder treatment success [106].

To overcome these challenges, innovative immune rejuvenation strategies are being explored, such as cytokine therapies (like IL-7 and IL-15) aimed at increasing the number and activity of functional T cells. Metabolic reprogramming efforts aim to enhance immune cell effector functions, thereby restoring their ability to target tumors. Combination treatments that include checkpoint inhibitors with senolytics (drugs targeting senescent cells), epigenetic modulators, or anti-inflammatory agents are also being investigated as comprehensive approaches to reverse immunosenescence and enhance immune surveillance [107].

These promising strategies hold promise for enhancing the effectiveness of immunotherapy in older cancer patients by targeting the core mechanisms of immune aging. Personalized treatments that restore both cellular and molecular immune functions are an essential step toward precision medicine for the aging population [108].

## Figures and Tables

**Table 1:** Comparative overview of age-related changes in the tumor microenvironment across major cancers.

Cancer Type	TME Feature	Key Pathways	Immune Changes	Clinical/Unique Aspect
Breast	Stromal fibrosis, adipose replacement	LOX, YAP/TAZ, TGF-β	↑Tregs, ↓naive T cells	Hormone receptor-positive, senolytics promising
Prostate	Chronic inflammation, senescent fibroblasts	NF-κB, AR dysregulation	↑Tregs, TAMs	CRPC development; androgen signaling altered
Lung	Fibrosis, epithelial senescence	STAT3, HIF-1α, EMT	Exhausted T cells, ↓naive T cells	Reduced immunotherapy efficacy
Bladder	ECM stiffening, recurrent inflammation	NF-κB, STAT3, LOX	↑MDSCs, ↑Tregs	Reduced BCG response in elderly
Colorectal	Dysbiosis, stromal fibrosis	TGF-β, IL-6, Wnt	↓mucosal immunity, ↑Tregs	Microsatellite instability-high tumors, microbiome-targeted therapy

Note: ↑ indicates an increase; ↓ indicates a decrease in the cell population.

## Data Availability

No new data were created or analyzed in this study. Data sharing is not applicable to this article.
